# Detection of renal allograft rejection using blood oxygen level-dependent and diffusion weighted magnetic resonance imaging: a retrospective study

**DOI:** 10.1186/1471-2369-15-158

**Published:** 2014-10-01

**Authors:** Guangyi Liu, Fei Han, Wenbo Xiao, Qidong Wang, Ying Xu, Jianghua Chen

**Affiliations:** Kidney Disease Center, First Affiliated Hospital, College of Medicine, Zhejiang University; Key Laboratory of Kidney Disease Prevention and Control Technology, Zhejiang Province; The Third Grade Laboratory under the National State Administration of Traditional Chinese Medicine, Hangzhou, China; Department of Nephrology, Qilu Hospital, College of Medicine, Shandong University, Jinan, China; Department of Radiology, First Affiliated Hospital, College of Medicine, Zhejiang University, Hangzhou, China

## Abstract

**Background:**

Acute rejection (AR) and acute tubular necrosis (ATN) are main causes of early renal allograft dysfunction. Blood oxygen level-dependent magnetic resonance imaging (BOLD MRI) and Diffusion weighted (DW) MRI can provide valuable information about changes of oxygen bioavailability and water diffusion by measuring R2* or apparent diffusion coefficient (ADC) respectively. We aimed to determine the value of BOLD MRI and DW MRI in detecting causes for early allograft dysfunction in renal allograft recipients.

**Methods:**

Fifty patients received renal allografts from deceased donors were analyzed, including 35 patients with normal renal function (control group), 10 AR patients and 5 ATN patients. Cortical R2* (CR2*) and medullary R2* (MR2*) were measured by BOLD MRI. Ten diffusion gradient *b* values (0, 5, 10, 20, 50, 100, 200, 400, 800, 1200s/mm^2^) were used in DW MRI. ADC values were measured in renal cortex (CADC) and medulla (MADC). CADC_l_ and MADC_l_ were measured under low *b* values (*b* ≤ 200 s/mm^2^), while CADC_h_ and MADC_h_ were measured under high *b* values (*b* > 200 s/mm^2^).

**Results:**

MR2* was significantly lower in AR group (18.2 ± 1.5/s) than control group (23.8 ± 5.0/s, p = 0.001) and ATN group (25.8 ± 5.0/s, p = 0.004). There was a tendency of lower levels on CADC_l_, MADC_l_, CADC_h_ or MADC_h_ in AR group than in control group. There were no differences on ADC values between AR group and ATN group.

**Conclusions:**

BOLD MRI was a valuable method in detection of renal allografts with acute rejection.

## Background

Kidney transplantation is the preferred treatment for most patients with chronic renal failure. Although advances in the skill of surgery and pharmacotherapy have led to improvement of the first-year renal graft survival, acute rejection (AR) and acute tubular necrosis (ATN) remain to be the main causes of early kidney allograft dysfunction [1]. Percutaneous allograft biopsy is the standard diagnostic method, but it is not suitable for dynamic monitoring due to the invasive nature. A non-invasive method is promising.

Blood oxygen level-dependent magnetic resonance imaging (BOLD MRI) is based on the paramagnetic properties of deoxyhemoglobin, which generates magnetic moments by its unpaired electrons in a magnetic field. The apparent relaxation rate denoted as R2* is proportional to the deoxyhemoglobin concentration. The increased R2* value implies increased deoxyhemoglobin level and decreased oxygen bioavailability in tissues [2]. Djamali *et al*. found BOLD MR images of renal allograft underwent AR had characteristic changes, which were related to the pathologic changes in renal allografts [3]. The previous study in our center found that in post-transplantation patients, R2* value in renal medulla significantly increased in ATN allografts and decreased in AR allografts compared with allografts with normal function, and these changes were reduced after the recovery of ATN or AR in dynamic follow-up [4].

Diffusion weighted (DW) MRI detects the change of Brownian motion of water protons in tissues and provides quantification of the change by calculating apparent diffusion coefficient (ADC) from DW images [5]. It simultaneously provides information on microcirculation perfusion. The microcirculation perfusion and water transportation are prominent physiological activities in kidney and the change of these activities may cause significant change of DW MRI signals. In transplanted rat kidneys, Yang *et al*. found that ADC values in renal cortex and medulla decreased significantly during angiotensin II-induced reduction in renal blood flow [6]. They also found allografts exhibited decreased ADC values while isografts exhibited similar ADC values compared with native kidneys [6]. Thoeny *et al*. found that ADC value was almost identical in the medulla and cortex of renal allografts in post-transplantation patients, while in human native kidneys ADC value was higher in the cortex than in the medulla [7].

Thus, BOLD MRI and DW MRI provide valuable information about the change of oxygen bioavailability and water diffusion in renal allografts. In this study we try to determine the value of BOLD MRI and DW MRI at 3 T in renal allograft recipients with early allograft dysfunction.

## Methods

### Study design

This is a retrospective study. The study protocols conformed to the provisions of the Declaration of Helsinki. The Ethic Committee of our hospital approved the protocols and all patients were informed and gave written consent. We included renal allograft recipients that received renal transplantation from deceased donors in our center and were willing to accept MRI examination between April 2010 and February 2011. The patients were scheduled to receive MRI 2–3 weeks after transplantation. The patients with AR or ATN received MRI within 3 days before or after percutaneous allograft biopsy. Totally, fifty patients’ data were analyzed. There were 35 patients who had stable normal renal function and no diagnosed episodes of AR or ATN during follow-up (control group), 10 AR patients (AR group) and 5 ATN patients (ATN group). The diagnosis of AR and ATN was confirmed by percutaneous allograft biopsy. In AR group, there were 6 patients with acute T-cell mediated rejection (TMR) and 4 patients with acute antibody-mediated rejection (AMR) according to Banff’05 criteria [8]. For ATN patients, the diagnosis was pathologically based on vacuolar degeneration or necrosis in diffuse or multifocal renal tubular epithelium and confirmed by clinical course of recovery without intensive immunosuppressive therapy such as methylprednisolone impulse, anti-T lymphocyte antibodies and plasma exchange. All these patients received combination of calcineurin-inhibitors, mycophenolate mofetil and prednisone as maintaining immunosuppression. Prednisone (10-15 mg) and mycophenolate mofetil (1.5 g) were administrated to the patients daily after 10 days post-operation. The dose of calcineurin-inhibitors such as tacrolimus and cyclosporin A was adjusted according to renal function and complications, and their trough blood concentration was monitored. For AR patients, we gave intensive immunosuppressive therapy such as methylprednisolone impulse, anti-T lymphocyte antibodies and plasma exchange only after renal biopsy and MRI. For ATN patients, no such intensive immunosuppressive therapy was prescribed. No angiotensin-converting enzyme inhibitors (ACEIs) or angiotensin II receptor blockers (ARBs) were used in any group. For hypertensive patients, we used calcium-channel blockers, β receptor blockers or clonidine to keep blood pressure below 160/100 mmHg.

All the patients were refrained from water or intravenous transfusion 4 hours before MRI and had no diuretic agents 12 hours before MRI. The mean arterial pressure (MAP) was measured right before MRI. During imaging, the patients had no oxygen inhalation and digital oxygen saturation was above 98%. The clinical results such as hemoglobin, serum creatinine and trough blood concentration of cyclosporin or tacrolimus were measured on the imaging day. The 24-hour urine output on the imaging day was recorded.

### MRI parameters

MRI was performed using a 3.0 Tesla system (GE Signa Horizon, Milwaukee, USA). For BOLD MRI, Echo-planar imaging sequence was performed to acquire images in coronal section during breath holds of 15 seconds. The parameters were as follows: repetition time 150 ms, TE 16 echo, echo time 2.5 ms, Flip angle 30 degree, Bandwidth ±31.25 kHz, Matrix 128 × 128, number of signals acquired 1, field of view 35 cm, thickness 6.0 mm, space 1.0 mm. Color map of R2* value was generated using software of T2* map in Functools 2 on MR working station. R2* values were measured using regions of interest (ROI) tool. Five to eight ROIs of the same size were placed in cortical region and in medullary region respectively, using regular T2 weighted image as reference. R2* measurements of all patients were completed by one radiologist unknowing the clinical or pathological data.

For DW MRI, Echo-planar imaging sequence was performed to acquire 16 DW images in transverse section. The parameters were as follows: repetition time 100 ms, echo time 2.5 ms, TE 65.5 ms, Flip angle 30 degree, Bandwidth ±31.25 kHz, Matrix 192 × 128, number of signals acquired 1, field of view 36 cm, thickness 6.0 mm, space 1.0 mm. The following 10 diffusion gradient *b* values were used: 0, 5, 10, 20, 50, 100, 200, 400, 800, 1200s/mm^2^. The images acquired under *b* value less than or equal to 200 s/mm^2^ were overlaid as the image of low *b* values, while the images acquired under *b* value greater than 200 s/mm^2^ were overlaid as the image of high *b* values. Color map of ADC value was generated using MADC software in Functools 2 on MR working station. According to the *b* values, the map was generated separately into ADC map of low *b* values (*b* value less than or equal to 200 s/mm^2^) and ADC map of high *b* values (*b* value greater than 200 s/mm^2^). ADC values were measured using regions of interest (ROI) tool. Five to eight ROIs of the same size were placed in cortical region and in medullary region respectively, using regular T2 weighted image as reference. ADC measurements of all patients were completed by one radiologist unknowing the clinical or pathological data.

### Data analysis

The analysis was performed by SPSS 16.0 software (SPSS Inc, Chicago, IL, USA). Numerical results were expressed as mean ± standard deviation, and were compared between groups using one-way analysis of variance (ANOVA) with POST-HOC test to perform pairwise multiple comparisons. The categorical data was expressed as counts or percentages and was compared using χ2 (Fisher’s exact test). Mann–Whitney test was used for nonparametric comparisons. Multinomial linear regression was used to determine the influence factors of R2* value or ADC value. Spearman correlation was used to determine the relation between R2* value and ADC value.

## Results

### Clinical characteristics

The clinical characteristics of patients in AR group, ATN group and control group were shown in Table 1. The patients in AR group received MRI in 31.1 ± 44.5 days (3 to 154 days) post-operation, later than that in control group (12.7 ± 6.4 days), while the patients in ATN group received MRI in 12.0 ± 5.3 days (7 to 21 days) post-operation. The patients in AR group and ATN group had higher levels of serum creatinine and MAP, and lower levels of hemoglobin and urine output compared with control group. All the patients in ATN group used tacrolimus, and their mean trough concentration level was lower than that in control group and AR group.

**Table 1:** **The clinical characteristics of patients in AR group, ATN group and control group**

Group	Control	AR	ATN
Case number	35	10	5
Age (years)	34.6 ± 10.1	36.3 ± 9.2	37.6 ± 11.5
Male: Female	28:7	6:4	4:1
Post-operation days	12.7 ± 6.4	31.1 ± 44.5^*^	12.0 ± 5.3
Post-biopsy days	NA	1.1 ± 3.9	1.4 ± 3.4
Urine output (l/24 hours)	2.36 ± 0.44	1.67 ± 0.96^*^	1.40 ± 0.66^*^
sCr (μmol/l)	88.8 ± 21.9	256.3 ± 173.5^*^	322.8 ± 179.1^*^
Hb (g/l)	104.2 ± 15.7	88.6 ± 15.1^*^	81.8 ± 14.4^*^
MAP (mmHg)	95.3 ± 10.9	109.9 ± 11.2^*^	107.7 ± 15.6^*^
Tacrolimus: CsA	30:5	6:4	5:0
Tacrolimus trough concentration (ng/ml)	7.5 ± 1.6	8.4 ± 2.4^#^	4.8 ± 1.6^*^
CsA trough concentration (ng/ml)	320.5 ± 64.8	238.6 ± 89.6	NA

Note: sCr, serum creatinine; Hb, hemoglobin; MAP, mean arterial pressure; CsA, cyclosporine A; NA, not applicable. ^*^p < 0.05 compared with the control group;^#^p < 0.05 compared with the ATN group.

### BOLD MRI data analysis

**Figure 1:**
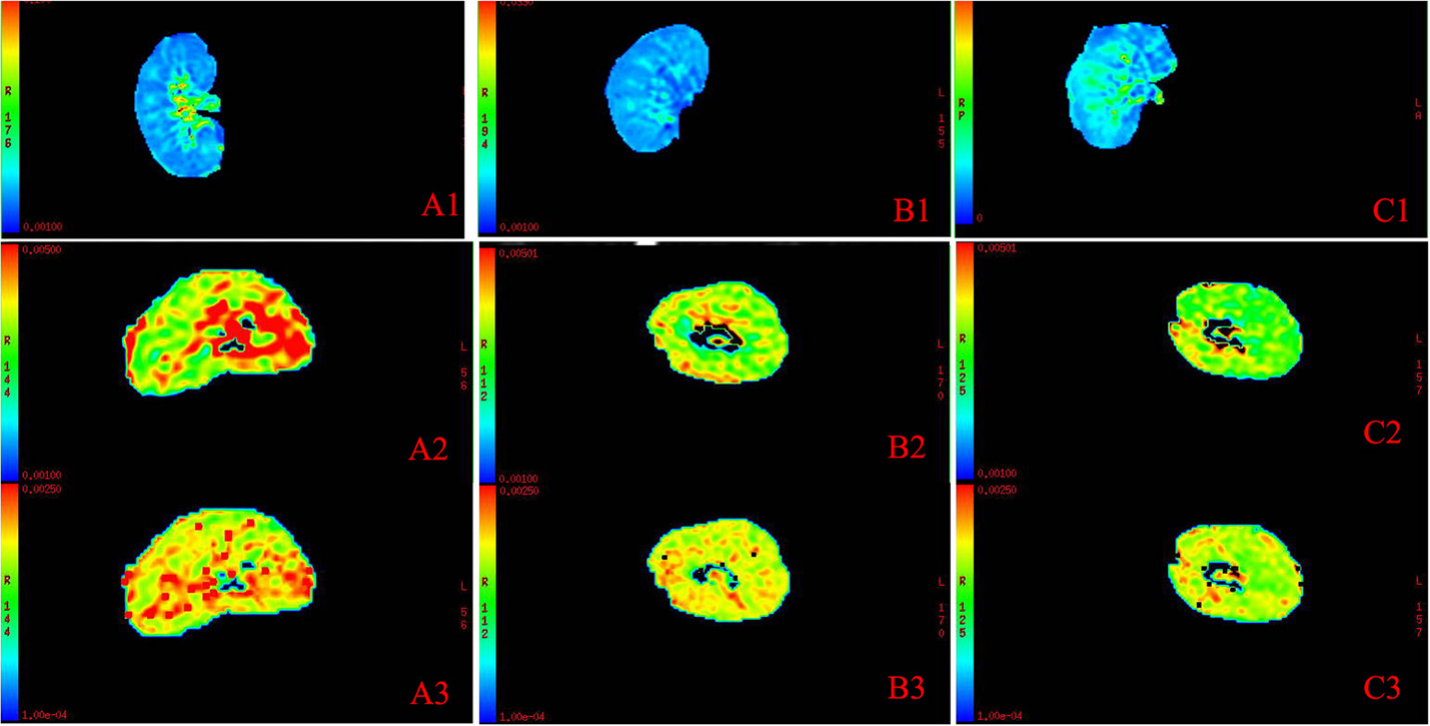
The representative R2* maps and ADC maps of renal allografts in AR group, ATN group and control group. **A**, the representative R2* map **(A1)** and ADC maps (**A2**, ADC map of low *b* values; **A3**, ADC map of high *b* values) of normal control allograft; **B**, the representative R2* map **(B1)** and ADC maps (**B2**, ADC map of low *b* values; **B3**, ADC map of high *b* values) of AR allograft; **C**, the representative R2* map **(C1)** and ADC maps (**C2**, ADC map of low *b* values; **C3**, ADC map of high *b* values) of ATN allograft. In R2* maps, the coronal section of the kidney is shown. The change of color from blue to green, orange, and then red represents the change of R2* value from lower to higher. There were more blue regions in the medulla of AR allograft **(B1)** and more green regions in the medulla of ATN allograft **(C1)**, compared with normal control allograft **(A1)**. In ADC maps, the transverse section of the kidney is shown. The change of color from green to orange, and then red represents the change of ADC value from lower to higher. There were less red regions and more green regions in AR allograft **(B2, B3)** and ATN allograft **(C2, C3)** compared with normal control allograft **(A2, A3)**, regardless of low or high *b* values.

The typical R2* maps of renal allografts in control group, AR group and ATN group were shown in Figure 1 and the average R2* values were shown in Table 2. In the color R2* maps of the coronal section of renal allografts, change of color from blue to green, orange, and then red represents the change of R2* value from lower to higher. In normal functioning allograft, renal cortex had the lowest R2* value, and the R2* value increased gradually from cortex to medulla. There were more blue regions in the medulla of AR allografts and more green regions in the medulla of ATN allografts, compared with those in normal allografts.

**Table 2:** The ADC and R2* values of renal allografts in AR group, ATN group and control group

Parameter	Control	AR	ATN
CR2* (1/s)	18.4 ± 4.4	16.6 ± 2.1	17.7 ± 3.7
MR2* (1/s)	23.8 ± 5.0	18.2 ± 1.5^*#^	25.8 ± 5.0
CADC_l_ (×10^-3^ mm^2^/s)	3.42 ± 0.36	3.04 ± 0.21^*^	2.98 ± 0.25^*^
MADC_l_ (×10^-3^ mm^2^/s)	3.46 ± 0.42	2.90 ± 0.32^*^	2.85 ± 0.25^*^
CADC_h_ (×10^-3^ mm^2^/s)	1.89 ± 0.23	1.53 ± 0.09^*^	1.72 ± 0.12
MADC_h_ (×10^-3^ mm^2^/s)	1.88 ± 0.28	1.53 ± 0.08^*^	1.69 ± 0.13

Note: CR2*, cortical R2*; MR2*, medullary R2*; ADC, apparent diffusion coefficient; CADC, cortical ADC; MADC, medullary ADC. The value of CADC_l_ and MADC_l_ were collected from the ADC map of low *b* values and the values of CADC_h_ and MADC_h_ were collected from the ADC map of high *b* values. ^*^p < 0.05 compared with the control group; ^#^p < 0.05 compared with the ATN group.

Medullary R2* (MR2*) value was significantly lower in AR group (18.2 ± 1.5/s) than that in control group (23.8 ± 5.0/s, p = 0.001) and ATN group (25.8 ± 5.0/s, p = 0.004). There were no significant differences on cortical R2* (CR2*) value among 3 groups. These results implied that the level of oxygen bioavailability in renal medulla increased in AR allografts compared with normal allografts and ATN allografts.

We did a follow-up BOLD MRI in 26 patients in control group at 34.5 ± 6.5 days post-operation. The paired-samples T test showed there were significant increase of serum creatinine (99.0 ± 23.7 μmol/l vs 88.0 ± 23.9 μmol/l, P = 0.004) and decreasing tendency of MR2* (22.0 ± 3.8/s vs 24.0 ± 5.4/s, P = 0.077) at the time of follow-up MRI compared with the first MRI (Table 3). However the MR2* value of the follow-up BOLD MRI in control group was higher than that in AR group (22.0 ± 3.8/s vs 18.2 ± 1.5/s, P = 0.0044).

**Table 3:** The comparison of R2* values obtained from the first BOLD MRI and the follow-up BOLD MRI in 26 patients in control group

	First MRI	Second MRI	P value
Post-operation days	10.5 ± 1.3	34.5 ± 6.5	
CR2* (1/s)	18.4 ± 5.1	16.9 ± 1.3	0.151
MR2* (1/s)	24.0 ± 5.4	22.0 ± 3.8	0.077
Serum Creatinine (μmol/L)	88.0 ± 23.9	99.0 ± 23.7	0.004

Note: CR2*, cortical R2*; MR2*, medullary R2*.

### DW MRI data analysis

The typical ADC maps of renal allografts in control group, AR group and ATN group were shown in Figure 1 and the average values of ADC were shown in Table 2. In the color ADC maps of the transverse section of renal allografts, change of color from green to orange, and then red represents the change of ADC value from lower to higher. In AR and ATN group, there were less red regions and more green regions both in the cortex and in the medulla of renal allografts compared with those in control group.

There was no significant difference between cortical ADC (CADC) value and medullary ADC (MADC) value in each group. The values of CADC_l_ and MADC_l_ were measured from ADC map of low *b* values (*b* value less than or equal to 200 s/mm^2^) and the values of CADC_h_ and MADC_h_ were measured from ADC map of high *b* values (*b* value greater than 200 s/mm^2^). The values of CADC_l_, MADC_l_, CADC_h_ and MADC_h_ in AR group were significantly lower than those in control group. Only the values of CADC_l_ and MADC_l_ in ATN group were lower than those in control group. But there were no differences on ADC values between AR and ATN group.

We did a follow-up DW MRI in 14 patients in control group at 34.6 ± 7.8 days post-operation. The paired-samples T test showed an increased serum creatinine level (98.1 ± 17.7 μmol/l vs 82.7 ± 21.0 μmol/l, P = 0.003) and no significant differences on CADC_l_, MADC_l_, CADC_h_ or MADC_h_ at the time of follow-up DW MRI compared with the first DW MRI (Table 4). However different from the results of the first DW MRI, in the follow-up DW MRI, there were no significant differences between CADC_l_, MADC_l_, CADC_h_ or MADC_h_ in control group and those in AR group (P = 0.15, 0.15, 0.09, 0.18 respectively).

**Table 4:** **The comparison of ADC values obtained from the first DW MRI and the follow-up DW MRI in 14 patients in control group**

	First MRI	Second MRI	P value
Post-operation days	10.6 ± 1.3	34.6 ± 7.8	
CADC_l_ (×10-3 mm2/s)	3.47 ± 0.28	3.29 ± 0.50	0.290
MADC_l_ (×10-3 mm2/s)	3.46 ± 0.25	3.21 ± 0.59	0.148
CADC_h_ (×10-3 mm2/s)	1.78 ± 0.15	1.78 ± 0.44	0.962
MADC_h_ (×10-3 mm2/s)	1.75 ± 0.18	1.78 ± 0.56	0.821
Serum Creatinine (μmol/L)	82.7 ± 21.0	98.1 ± 17.7	0.003

Note: ADC, apparent diffusion coefficient; CADC, cortical ADC; MADC, medullary ADC. The value of CADC_l_ and MADC_l_ were collected from the ADC map of low *b* values and the values of CADC_h_ and MADC_h_ were collected from the ADC map of high *b* values.

### The influence factors of R2* value or ADC value

Multinomial linear regression analysis revealed that clinical characteristics including age, post-operation days, post-biopsy days, levels of serum creatinine, hemoglobin, urine output and MAP were not the independent influence factors for CR2*, MR2*, CADC_l_, MADC_l_, CADC_h_ or MADC_h_. Spearman correlation analysis revealed that there were positive correlation between MR2* and CADC_h_ (r = 0.4, p = 0.004), and MR2* and MADC_h_ (r = 0.332, p = 0.018).

## Discussion

The current study showed MR2* value was significantly lower in AR allografts compared with normal allografts and ATN allografts. In BOLD MRI, decreased R2* value implied increased oxygen bioavailability in tissues [2], so decreased MR2* value in AR allografts indicated that the level of oxygen bioavailability increased in the medulla of AR allografts. One reason may be the decreased glomerular filtration rate (GFR) in AR episodes, which causes decreased production of original urine and then less tubular reabsorption, and subsequent reduced oxygen consumption. Another possibility is the change of oxygen delivery by blood supply. Wentland *et al*. used MRI to measure renal cortical and medullary perfusion, oxygen bioavailability in the kidneys of porcine models and found that R2* values decreased in renal cortex and medulla when the local blood perfusion increased [9]. Acute rejection causes obvious inflammation in renal medulla, which may increase blood shunting to the medulla [10]. However, recent studies using perfusion MRI revealed that blood perfusion both in renal cortex and in medulla decreased significantly in rejected allografts compared with that in normal allografts [11, 12]. So decreased oxygen consumption may be the main cause for increased oxygen bioavailability in renal medulla. Clinically, BOLD MRI has been applied in a few studies that showed MR2* values were significantly decreased in AR allografts compared with those in normal allografts [3, 4, 11, 13]. Djamali *et al*. found changes of BOLD MRI were related to the pathologic changes in AR allografts, and AR allografts with vascular injury such as type IIA or peritubular capillary C4d positive AR had the lowest MR2* level [3].

DW MRI provides information of water diffusion and microcirculation perfusion at the same time. In human native kidneys, CADC values were higher than MADC values [7, 14], however in renal allografts there were no significant differences between CADC and MADC [7]. The *b* value is the sensitive coefficient of diffusion and the higher *b* value represents more sensitive imaging of diffusion. Under low *b* values, ADC values are influenced by both diffusion and blood perfusion; while under high *b* values, the influence of blood perfusion is avoided [14]. In the current study, both ADC_l_ and ADC_h_ values were lower in AR allografts and only ADC_h_ values were lower in ATN allografts, compared with those in normal allografts. Perfusion MRI proved that there was significant decrement on renal perfusion in AR allografts but not in ATN allografts [10, 11]. The current results supported that both levels of water diffusion and blood perfusion were impaired in AR allografts, whereas only level of water diffusion was impaired in ATN allografts.

Recently, a few studies evaluated the diagnostic value of DW MRI in acute renal allograft dysfunction [15, 16]. One study compared renal allografts under AR(n = 10), ATN (n = 7) and immunosuppressive toxicity(n = 4) with 49 patients with stable renal allograft function [15]. They found that ADC values of allografts under ACR, ATN and immunosuppressive toxicity, respectively, were significantly lower than those of normal controls, but there were no significant differences in ADC values among ACR, ATN, and immunosuppressive toxicity groups [15]. Another study performed DW MRI in 15 renal allograft recipients including 10 with stable function and 5 with renal dysfunction (4 AR and 1 ATN) [16]. They separately analyzed ADC values by the influence of diffusion or microcirculation perfusion, and found that ADC values due to perfusion decreased to less than 12% in allografts with renal dysfunction and were correlated with the level of creatinine clearance [16].

Presently most kidney MRI studies were performed at 1.5 T. Higher field strengths may have the advantages such as higher signal-to-noise ratios, faster imaging and better spatial resolution [17]. Park *et al*. performed BOLD MRI at 3 T in 8 normal functioning renal allografts and 4 AR allografts, and found MR2* values were significantly lower in AR allografts than in normal allografts at different gradient echo of 8, 16 or 20[18]. Lanzman *et al*. performed DW MRI at 3 T in 40 kidney recipients, divided into one group with good or moderate kidney function (GFR > 30 ml/min/1.73 m^2^, n = 23) and the other group with impaired kidney function (GFR ≤ 30 ml/min/1.73 m^2^, n = 17) [19]. They found both CADC and MADC values were significantly lower in allografts with impaired kidney function, and another parameter named fractional anisotropy of renal medulla was significantly lower in recipients whose kidney function did not recover in 6 months than in those with stable kidney function [19]. There were no reports about the comparative effects between 1.5 T and 3 T in kidney imaging now. However, a study performing BOLD MRI of parotid glands at 1.5 T or 3 T for the same group of patients was reported [20]. It showed that BOLD MRI at 3 T, but not at 1.5 T, was able to detect changes of R2* value during gustatory stimulation, which was consistent with an increase in oxygen consumption during saliva production [20].

This study had some limitations. First, although we restricted water or intravenous transfusion 4 hours before MRI and diuretic agents 12 hours before MRI, we did not measure the exact hydration status in individual patients before MRI. Commonly, there was water retention in patients with impaired glomerular filtration such as AR or ATN patients. This may lead to decreased MR2* [21], while different hydration states do not significantly influence ADC values [22]. Second, the time of MRI in AR patients was later than patients with normal renal function and ATN patients. The R2* values and ADC values may change following the recovery of renal allografts such as improvements in tissue edema and inflammation. However, we did a follow-up BOLD MRI in 26 patients with normal renal function at 34.5 days after operation, the same as that in AR group. A negative correlation betweenMR2* and days post-operation was observed, however, the MR2* value in the follow-up MRI was 22.0 ± 3.8/s, higher than that in AR patients (18.2 ± 1.5/s). We also did a follow-up DW MRI in 14 patients in control group and found no differences on ADC values between the first DW MRI and the follow-up DW MRI. However, there were no significant differences on ADC values between the follow-up DW MRI in control group and that in AR group. Due to the relatively small P values obtained, the reason may be the limited cases at the time of follow-up DW MRI in control group. Third, this was a retrospective analysis in one center with limited cases. The patients in control group were not subjected to renal biopsy to evaluate possible subclinical rejection. A well-designed perspective study is necessary to prove the diagnostic values of BOLD MRI and DW MRI in renal allografts.

## Conclusions

BOLD MRI was a valuable method in detection of renal allografts with AR, and its diagnostic values need to be proved by a well-designed, large, perspective study.
